# Musculoskeletal Ultrasound to Identify Subclinical Joint and Periarticular Involvement in Patients With Inflammatory Bowel Disease: A Systematic Literature Review

**DOI:** 10.3389/fmed.2022.919521

**Published:** 2022-05-16

**Authors:** Garifallia Sakellariou, Annalisa Schiepatti, Davide Scalvini, Francesca Lusetti, Erica Fazzino, Federico Biagi, Carlomaurizio Montecucco

**Affiliations:** ^1^Istituti Clinici Scientifici Maugeri IRCCS, Pavia, Italy; ^2^Department of Internal Medicine and Medical Therapy, University of Pavia, Pavia, Italy; ^3^Gastroenterology Unit, Istituti Clinici Scientifici Maugeri IRCCS, Pavia, Italy; ^4^Chair and Division of Rheumatology, IRCCS Policlinico San Matteo Foundation, Pavia, Italy

**Keywords:** arthritis, disability, inflammatory bowel disease, imaging, ultrasonography, systematic literature review

## Abstract

**Background:**

Musculoskeletal ultrasonography identifies subclinical joint and entheseal inflammation, and it might be of value in patients with inflammatory bowel diseases (IBD), which are at higher risk of inflammatory arthropathy and disability. Our aim was to retrieve the evidence on the applications of ultrasound in patients with non-arthropathic IBD.

**Methods:**

Studies enrolling patients with IBD without arthritis, undergoing ultrasound of joints, tendons or entheses were eligible. The outcomes of interest encompassed the frequency of ultrasound-detected lesions, their accuracy in diagnosing arthritis, their prognostic role and sensitivity to change. All study types, excluding case reports, case series and narrative reviews, were included. Search strategies were applied in PubMed and Embase. Abstract and full-texts were evaluated by pairs of reviewers. The risk of bias was evaluated through the Newcastle-Ottawa scale or the Quality Assessment of Diagnostic Accuracy Studies (QUADAS) 2. The protocol was registered in PROSPERO (CRD42021264972).

**Results:**

Out of 2,304 records, eight studies were included, all reporting the frequency of lesions, while only three evaluated also the diagnostic accuracy. All studies had a cross-sectional design, with no evidence on prediction or follow-up. All studies evaluated the entheses, while only three the joints. The most common chronic lesions were entheseal thickening (up to 81.5%) and enthesophytes (67.9%), while entheseal erosions were present in 16%−17% of patients. Among inflammatory lesions, power Doppler was reported in 14%−67% of patients. There were no differences among Crohn's disease or ulcerative colitis and depending on disease activity, while there were contrasting results on different disease durations. When evaluating the diagnostic performance, the best specificity for a diagnosis if IBD was 0.88 (95%CI, 0.8–0.94) for joint abnormalities. Also, the best sensitivity was 0.88 (95%CI, 0.76–0.95) for entheseal lesions. No studies assessed of the combination of lesions. Due to the limited number of studies, meta-analyses were not performed.

**Conclusions:**

Despite the possible value of ultrasound in IBD, there is limited evidence deriving from cross-sectional studies. Longitudinal studies are needed to clarify the role of this technique, while its current placement might be that of complementing clinical assessment, in particular in early intestinal disease.

## Introduction

Inflammatory bowel diseases (IBD), which include Crohn's disease (CD) and ulcerative colitis (UC), are common chronic inflammatory diseases of the gastro-intestinal tract characterized by unknown etiology and heterogeneous clinical manifestations, both intestinal and extra-intestinal (1–4). The key diagnostic features of UC include diffuse mucosal inflammation extending proximally from the rectum, whereas in CD patchy and segmental transmural inflammation can occur in any site of the gastrointestinal tract (1–4). Among the extra-intestinal manifestations of IBD, inflammatory arthritis, pertaining to the group of spondyloenthesoarthritis (SpA), is undoubtedly the most common, with an estimated prevalence ranging from 13 to 39% of all IBD patients (5–11). The clinical phenotypes of IBD-associated SpA include peripheral arthritis and axial manifestations related to sacroiliitis with or without concomitant spondylitis, and imply a chronic joint involvement and increasing disability (12). Musculoskeletal symptoms leading to the diagnosis of SpA usually develop after the diagnosis of IBD, but in up to 20% of patients rheumatological involvement precedes the gastrointestinal symptoms and leads to the diagnostic suspicion of IBD (7, 13, 14).

In the last years, clinical interest has been dedicated to IBD patients who have undiagnosed SpA (5, 15), reflecting the promising results achieved in the similar field of patients with psoriasis (16). In fact, in patients affected by psoriasis without joint involvement, imaging-detected inflammation of joints and periarticular structures significantly predicted the subsequent development of arthritis (17).

Ultrasound assessment of entheseal and joint sites has been recognized as a powerful and reliable tool to evaluate subclinical joint involvement (15). In fact, ultrasound has shown a greater accuracy to identify musculoskeletal inflammation, compared to clinical evaluation (18), and this might even be of greater relevance in patients with IBD, as some immunosuppressive treatments might mask an underlying joint involvement. However, little is known on the prevalence of occult SpA in IBD patients and the diagnostic and prognostic relevance of ultrasonographic articular/enthesal findings in this subgroup of patients.

The aim of the present systematic literature review is to evaluate the available evidence on the prevalence of ultrasonographic abnormalities in IBD patients without a previous history of inflammatory arthritis and their diagnostic and prognostic role.

## Methods

The SLR was conducted following the PRISMA 2020 Checklist (19). The target population consisted of patients with a diagnosis of IBD and no previous diagnosis of inflammatory arthritis. Five clinical questions were identified, in order to drive the searches and the inclusion of the articles. The areas of interest encompassed the frequency of ultrasound-detectable abnormalities in the joints and tendons, the diagnostic performance of ultrasonographic variables in the diagnosis of arthritis, with clinical diagnosis as reference standard, the prognostic value of ultrasonographic findings in identifying patients at risk of development of arthritis, and the value of ultrasound in monitoring abnormalities. The research questions were transformed into the Patients, Intervention, Comparator, Outcome, Study Type (PICOs) format (Table 1), sharing pre-defined inclusion and exclusion criteria. Moreover, we planned subgroup assessments for each research question, comparing CD and UC, patients with and without arthralgia, patients with mechanical and inflammatory arthralgia, patients with active and inactive IBD, patients with different disease duration of IBD, patients with joint symptom duration of less or more than 12 months. The protocol of the SLR was shared among authors and registered in the PROSPERO database (registration number CRD42021264972).

**Table 1:** Research questions and corresponding PICOs, driving the literature search and the inclusion/exclusion of the articles. Population and Intervention are the same for all research questions.

**Research question**	**Population**	**Intervention**	**Comparator/Reference standard**	**Outcome**	**Study type**
*What is the frequency of abnormalities, detected in the joints and in periarticular structures by ultrasonography, in patients with IBD without a diagnosis of arthritis?*	Adult patients with IBD without a diagnosis of arthritis	Musculoskeletal US of joints and tendons, including entheses	Not required	Frequency of US abnormalities	Longitudinal or cross-sectional cohort studies, case-control studies, randomized clinical trials, systematic literature reviews, meta-analyses, diagnostic accuracy studies, case series
*What is the value of ultrasonographic findings in making a diagnosis of arthritis in patients with IBD without a diagnosis of arthritis?*			Clinical diagnosis of arthritis	Diagnostic accuracy: sensitivity, specificity, AUC, diagnostic Odds Ratio, LR+, LR–, PPV, NPV	Longitudinal or cross-sectional cohort studies, case-control studies, randomized clinical trials, systematic literature reviews (in order to review the references), meta-analyses, diagnostic accuracy studies, case series
*What is the prognostic value of ultrasonographic findings against the development of arthritis in patients with IBD without a diagnosis of arthritis?*			Other predictors of arthritis (not required)	Development of arthritis: OR, RR, HR	Longitudinal cohort studies, case-control studies, systematic literature reviews (in order to review the references), meta-analyses, case series
*What is the value of ultrasonography in monitoring lesions in the joints and periarticular structures in patients with IBD without a diagnosis of arthritis?*			Other means (clinical assessment, other imaging) to monitor the joints (not required)	Sensitivity to change	Longitudinal cohort studies, case-control studies, systematic literature reviews (in order to review the references), meta-analyses, case series

Search strategies were applied to PubMed and Embase by one author (GS; January 1^st^ 1980–July 29^th^ 2021; Supplementary Table S1). The time interval was chosen to include all studies since the introduction of musculoskeletal ultrasound. The records retrieved were transferred into a bibliographic management software (Zotero) and duplicates removed. Four investigators (EF, FL, DS, AS) performed screening, selection, data extraction and Risk of Bias (RoB) assessment, working in pairs to assess titles and abstracts to define eligibility for detailed review. Full texts of the included records were retrieved, and eligibility for final inclusion was assessed. Disagreement was resolved by discussion within the pairs and, further, by involving a fifth reviewer (GS). Data from the included articles were extracted in pre-specified forms, including general information on the article, features of the population and, when available, 2 × 2 tables of diagnostic accuracy, 2 × 2 contingency tables, Odds Ratios or Risk Ratios. The references of the included studies were screened to look for further eligible articles. The RoB of the studies included only in the analysis on the prevalence of abnormalities was assessed with the Newcastle-Ottawa scale (NOS) for cohort and case-control studies (20), while studies included in the diagnostic question were evaluated through the Quality Assessment of Diagnostic Accuracy Studies (QUADAS-2) tool for diagnostic studies (21). Results were presented in summary of evidence tables. Diagnostic accuracy meta-analyses could be considered in case data on a single variable were available from at least four clinically homogeneous studies. Summary graphs reporting sensitivities and specificities were created with Review Manager (RevMan) Version 5.4, The Cochrane Collaboration, 2020.

## Results

**Figure 1:**
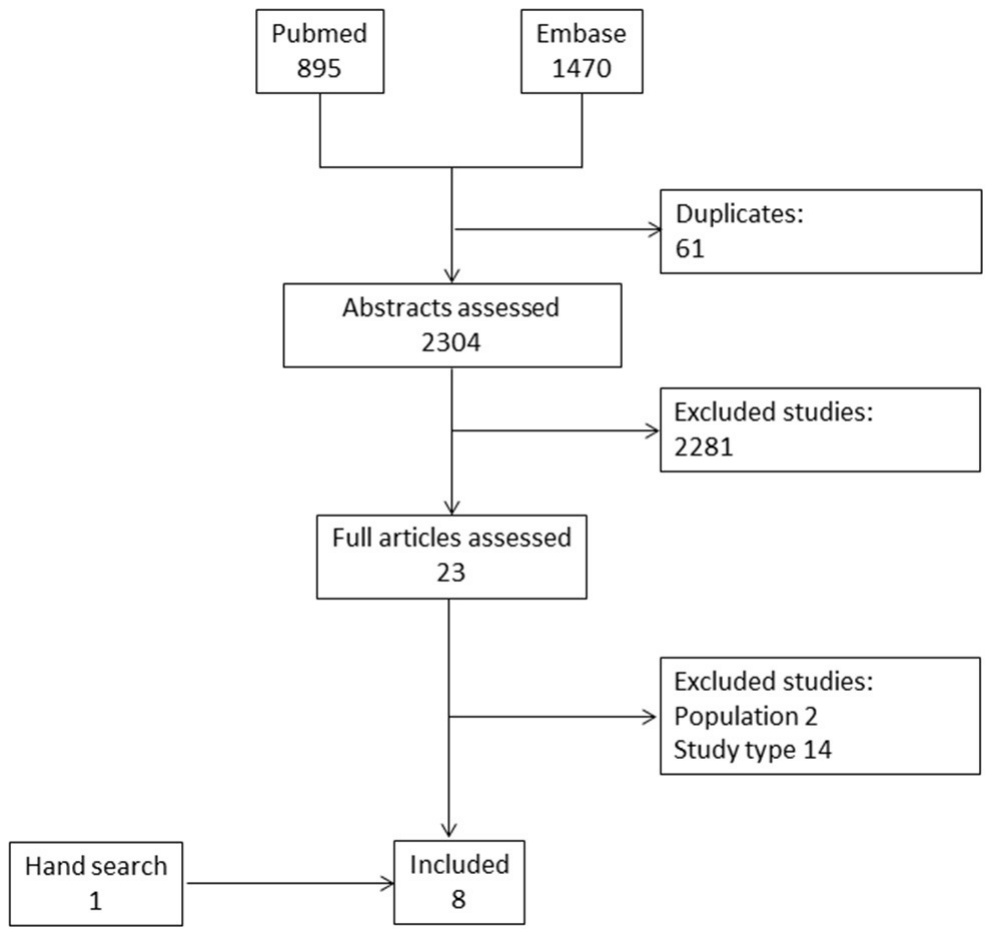
Flow-chart.

Of 2,304 abstracts evaluated, eight studies were finally included (15, 22–27). Of those, seven articles were retrieved from the electronic databases and one by hand search (Figure 1) (28). The total number of included patients was 679. All of these studies allowed to derive information on the frequency of lesions, while only three studies presented data on the diagnostic accuracy of ultrasonographic findings to identify patients with arthritis among patients with IBD (22, 24, 25). Three studies had a case-control design (15, 23, 24), while the remaining were cross-sectional studies. The absence of prospective studies, therefore, did not allow to retrieve any evidence on the value of musculoskeletal ultrasound to define prognosis and to monitor joint and entheseal lesions. All of the included studies assessed various entheseal sites (Table 2), while only three included also an evaluation of joints (22, 26, 28). A single study reported scanning synovial tendons (22). In particular, the quadriceps tendon, the proximal and distal patellar tendons, the Achilles tendon and plantar fascia were assessed in all of the studies, the insertion of the common extensor tendon at the epicondyle in 4 studies (22, 24, 25, 28), the triceps tendon (27) and the insertion of the common flexor tendon at the medial epicondyle in one study each (28). Among the joints, the metacarpophalangeal joints (MCP) (28), the metatarsophalangeal joints (MTP) (26) were evaluated in one study, while the knees (22, 26) and the ankles (22, 26) were evaluated in two studies each.

**Table 2:** Summary of frequencies for each entheseal lesion.

	**Any lesion**	**Chronic lesions**	**Acute lesions**	**Increased thickness**	**Hypoechogeni** **city**	**Power Doppler**	**Bursitis**	**Erosions**	**Entesophytes**	**Calcifications**
**All entheses**	30^*^-87.9	83–83.8	31–43.8	81.5	–	14–67	27.1	15–16	67.9	–
CD	83.8	79.4	42.2	–	–	21.5	–	–	–	–
UC	90.2	87	45.3	–	–	31.6	–	–	–	–
**Triceps tendon**	–	–	–	73.3^*^	0^*^	0^*^	–	0^*^	0^*^	0^*^
**Quadriceps**	–	–	–	5.71^*^-40.7^*^	10.5^*^	2.3^*^-2.5	0–7.86^*^	0^*^-2.5	0^*^-38.2	0^*^
**Proximal patellar**	–	–	–	8.57^*^-42	4.7^*^	0–3.5^*^	0	0^*^-3.7	0^*^-2.4	1.2^*^
**Distal patellar**	–	–	–	6.43^*^-58	19.8^*^	3.7–16.3^*^	0^*^-21	0^*^-3.7	0^*^-7.4	1.2^*^
**Achilles tendon**	–	–	–	0.71^*^-23.3^*^	33.7^*^	0–20.9^*^	2.14^*^-7.4	0^*^-1.2	1.4^*^-56.8	2.3^*^
**Plantar fascia**	–	–	–	0^*^-16	1.2^*^	1.2	0	0^*^-2.5	0^*^-3.7	0^*^

*Results are presented as percentages and ranges, when available*;

* *proportion of entheses; when not specified percentages refer to the proportion of patients*.

Six studies applied semi-quantitative scoring systems to assesses entheses, in particular the Glasgow Ultrasound Enthesitis Scoring System (GUESS) (15, 22, 23, 25) and the Madrid Sonographic Enthesitis Index (MASEI) (22, 24, 25) were adopted by four and three studies, respectively. The use of high-end ultrasound equipment was reported by five studies (22, 24–27), all of the studies were performed after 2010, which likely implies technically comparable equipment. Three studies presented comparative data in CD and UC (15, 22, 26), while two studies compared active and inactive disease (15, 24). Information stratified based on IBD disease duration was obtained by three studies (15, 22, 27), while no studies addressed the influence of the presence of arthralgia, the type of arthralgia and the duration of joint symptoms.

The complete summary of findings of the included studies is reported in the Supplementary Tables S2, S6, in the online only supplement. The summary of the assessment of the Risk of Bias is shown in Table 3.

**Table 3:** Assessment of the risk of bias. Newcastle-Ottawa Scale: each asterisk refers to the fulfillment of the items of the different components of the scale. QUADAS-2: green refers to a low risk of bias, yellow to unclear risk of bias and red to high risk of bias.

*What is the frequency of abnormalities, detected in the joints and in periarticular structures by ultrasonography, in patients with IBD without a diagnosis of arthritis? Newcastle-Ottawa Scale*				
**Study**	**Selection**	**Comparability**		**Outcome/exposure**
Bandinelli et al. (15)	***	**		***
Hsiao et al. (23)	*	*		***
Rodriguez-Caminero and Queiro (28)	****	*		*
Rovisco et al. (26)	***	*		*
Ureyen et al. (27)	***	*		*
*What is the value of ultrasonographic findings in making a diagnosis of arthritis in patients with IBD without a diagnosis of arthritis? QUADAS-2*				
**Study**	**Selection**	**Test**	**Standard**	**Flow/Timing**
Bertolini et al. (22)				
Martinis et al. (25)				
Husic et al. (24)				

### Frequency of Ultrasound-Detected Abnormalities

All of the eight included studies allowed to retrieve information on the prevalence of ultrasound-detectable lesions. The characteristics and results of the included studies are reported in Supplementary Table S2. Among the studies assessing entheseal involvement, four evaluated the presence of bone erosions, three entheseal thickening, enthesophytes and power Doppler, two evaluated bursitis, while a single study reported the prevalence of calcifications and hypoechogenicity.

**Figure 2:**
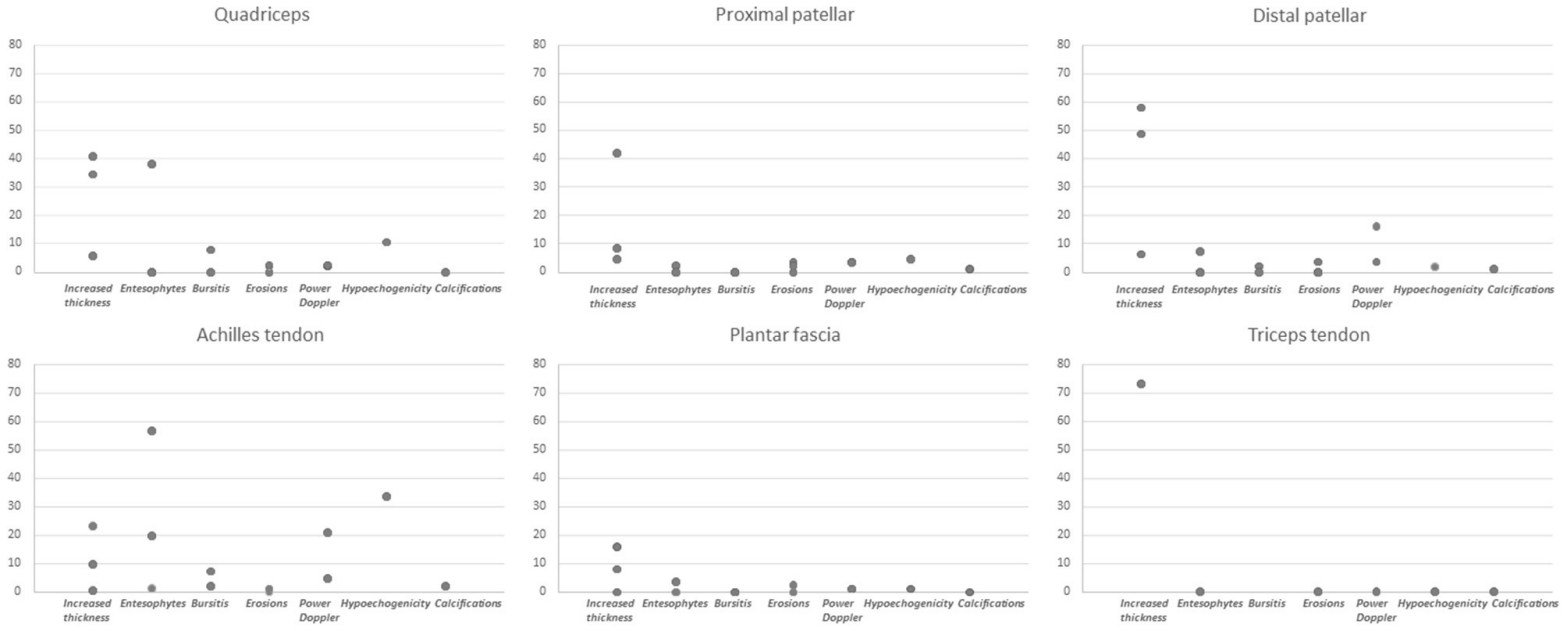
Summary of frequencies for each lesion at entheseal sites.

The range of frequencies retrieved from the studies for each lesion is reported in Table 2 and Figure 2.

Among the tested structures, entheseal involvement emerged as the most frequent lesion, with an overall range from 30 to 87.9% across studies. Specifically, chronic lesions were consistently found with a frequency ranging from 83 to 83.3%, while 31%−43.8% of entheses showed signs of acute inflammation. Joint involvement was reported with a lower frequency, from 19.7 to 48.8%. At entheseal level, among abnormalities in gray scale (GS), increased thickness was reported in 81.5% of patients, entesophytes in 67.9% and erosions with a frequency ranging from 16 to 17%, while bursitis was described in 27.1% of patients. The frequency of power Doppler (PD) was widely variable across studies, ranging from 14 to 67%.

When analyzing specific sites, the only lesion reported at the triceps tendon was increased thickness, while at the remaining sites for which frequency data were available in detail (quadriceps tendon, proximal and distal patellar tendon, Achilles tendon and plantar fascia) all lesions were assessed. Frequencies were reported based on the total number of patients in some studies (15, 22, 24–26, 28), while in some others on the number of assessed entheses (23, 27). Specifically, increased thickness was more frequently reported at the distal patellar tendon (range 6.43% of entheses to 58% of patients), hypoechogenicity was found more frequently at the Achilles tendon (33.7% of sites), as well as PD (from 0% of patients to 20.9% of sites), enthesophytes (from 1.42% of sites to 56.8% of patients) and calcifications (2.3% of sites). Bone erosions had a limited frequency at specific sites, with a maximal frequency of 3.7% of patients at the proximal and distal patellar tendon insertion. The frequencies for each lesion are displayed in Figure 2.

In studies comparing patients with CD and UC, no significant differences in terms of frequency of entheseal or joint involvement among diseases emerged (Supplementary Table S3) (15, 22, 26). Disease activity did not seem to be related to ultrasonographic findings: in fact, no association was found between clinical activity of IBD and entheseal involvement defined by MASEI (24) or GUESS (15), the presence of PD (15, 24), erosions and enthesophytes (Supplementary Table S4) (24). The evidence on the impact of disease duration, instead, was more contrasting. In fact, while two studies reported no differences in GUESS and PD (15, 27), a recent study reported a higher prevalence of entheseal abnormalities in patients with more than 1 year of IBD disease duration (90% vs. 72%, *P* = 0.003), and this applied particularly to bone erosions (7.4% vs. 0%, *P* = 0.04), while GUESS and MASEI did not significantly differ (Supplementary Table S5) (22).

Joint and tenosynovial involvement were less frequently assessed. A single study included synovial tendons in the scanning protocol, without however reporting the results of the assessment (22), while details on the prevalence of joint involvement were reported by two studies, with a frequency of 19.7 and 48.8%, depending on the sites (22, 26). The methodological quality of the included studies, assessed through the NOS, was mostly adequate for patient selection and comparability, while it was lower for outcome assessment in three studies.

### Value of Ultrasound-Detected Lesions in Making a Diagnosis of Arthritis

Of the three studies reporting data on the diagnostic accuracy to detect arthritis, one had a case-control design (24), all described entheseal lesions (22, 24, 25), while a single study reported information on joint involvement (22). Of note, all of the studies were published after 2020, when a shared definition of enthesitis, proposed by the Outcome Measures in Rheumatology (OMERACT), was already available (29).

In detail, Bertolini et al. enrolled 148 consecutive patients with IBD, of which 27 were treated by biological drugs, assessing 12 entheseal sites to derive MASEI and GUESS, as well as synovitis and tenosynovitis at the knees and ankles. Husic and colleagues assessed 14 entheseal sites, in order to apply a modified version of MASEI, in 47 patients with IBD and 44 healthy controls. Finally, Martinis et al., evaluated a cohort of 158 IBD patients, with a median disease duration of about 10 years, assessing 12 entheseal sites to calculate MASEI.

**Figure 3:**
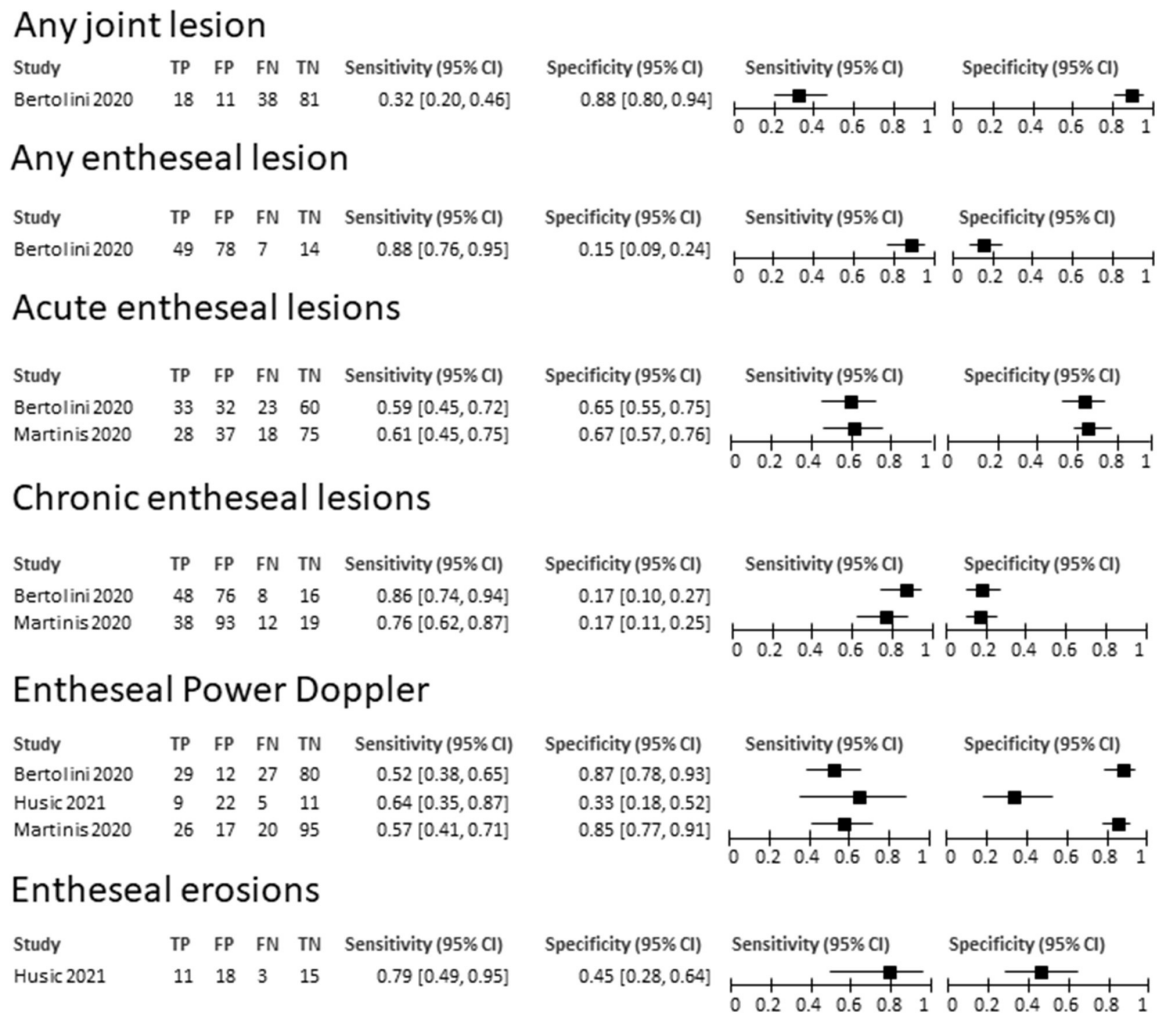
Summary of sensitivities and specificities of ultrasound-detected lesions for a diagnosis of arthritis in patients with IBD. TP, true positive; FP, false positive; FN, false negative; TN, true negative.

The limited number of available studies did not allow any quantitative synthesis of the results. The features and findings of the included studies are summarized in Supplementary Table S6. The highest specificity for the detection of arthritis was provided by the overall presence of any joint abnormalities (specificity, 0.88, 95% CI 0.80–0.94), while the highest sensitivity by any entheseal lesion (sensitivity 0.88, 95% CI 0.76–0.95), although at the cost of a low specificity. Also, chronic entheseal lesions and erosions had a good sensitivity, however no single lesion or combination of lesions achieved an adequate compromise between sensitivity and specificity. The sensitivities and specificities of the primary studies are summarized in Figure 3. The RoB of the included studies, assessed through the QUADAS2 tool, resulted to be low for two studies and high in one for selection, unclear for the test in all studies, and low for reference standard and flow and timing in all studies.

## Discussion

This systematic review provides an updated overview on the clinical applicability of musculoskeletal ultrasonography in patients with IBD without an overt joint involvement. In the field of rheumatology, ultrasonography has gained increasing success in the last two decades in light of the technical advances, the easy availability in an outpatient setting allowing an immediate application of the results to patient management, low cost and good acceptability (30). Ultrasonography has been proven to be more sensitive than clinical examination in identifying synovitis (31), and more specific than clinical examination in identifying entheseal involvement (32). For these reasons, ultrasonography has been considered an interesting imaging technique to evaluate patients at higher risk of developing arthritis, particularly in the field of psoriasis (17, 33), where a predictive value over the future development of arthritis has been demonstrated (34). While the amount of evidence for the application in psoriatic patients is already significant, with ongoing large prospective studies (16), in the field of IBD the interest on ultrasound is more recent.

We retrieved a total of 8 studies, all published after 2011, reflecting the growing and still evolving interest on this possible application of ultrasound. The main results pertain to the area of prevalence of ultrasound lesions, with some evidence also on their diagnostic value.

More precisely, we found a high variability in the frequency of both chronic and acute lesions in patients with IBD, in particular the overall prevalence of entheseal abnormalities, of entheseal PD as well as that of joint abnormalities were reported with a wide range across studies. This great heterogeneity could be related to populations under investigation, which largely differed, in terms of inclusion criteria, disease duration, clinical setting and type of treatment. The fact that the prevalence of lesions was calculated in some cases by using the number of patients and in others the number of entheses as statistical unit should be regarded as a possible further source of heterogeneity. The high degree of heterogeneity, however, seems to be in line with that found in patients with psoriasis and psoriatic arthritis (33).

As far as the gastroenterological setting is concerned, we found that only IBD disease duration correlated with a higher frequency of ultrasound abnormalities; however, this result emerged from a single study which enrolled patients with a very short disease duration (<12 months of disease duration). In this paper, patients with IBD from more than 1 year had a higher number of abnormal entheses and more entheses presenting bone erosions, compared to the early patients. Although it is known that articular manifestations in IBD patients can precede the onset of gastrointestinal symptoms, papers that evaluated the risk of developing arthritis after the diagnosis of IBD are scarce (1, 2, 5, 12).

On the other hand, we did not find any correlation between the type of IBD and the disease activity. Similarly, the remaining subgroup analyses did not provide any relevant result.

The three studies reporting information on the diagnostic accuracy allowed to retrieve data on the performance of single lesions, once again showing inconsistent results, with no information on the impact of a combination of lesions. None of the tested ultrasound-detectable lesions showed an acceptable compromise between sensitivity and specificity, although the limited number of included studies does not allow to draw solid conclusions. The highest sensitivity (0.88) was achieved considering any possible entheseal abnormality, at the cost of a poor specificity. The highest specificity, instead, was achieved by chronic entheseal lesions, with a range of specificities from 0.76 to 0.86. Given the paucity of studies, a quantitative summary of the results by a diagnostic accuracy meta-analysis was not possible. Once again, the lack of studies testing a combination of elementary lesions in cohorts reproducing a realistic clinical setting has already been described as a characteristic limitation of ultrasonographic studies in rheumatology, and represents a relevant issue to be addressed in future research (18, 35).

A major intrinsic limitation of our study is represented by the fact that most of the studies focused on the assessment of entheses, with limited information on the joints and no information on tenosynovitis. While in spondyloenthesoarthritis enthesitis has been identified as the primary lesion characterizing the disease process, tenosynovitis is emerging as a possible early lesion in new-onset peripheral inflammatory pain (36), and its low prevalence in healthy subjects suggests specificity for arthritis (37). In addition, tenosynovitis was the only lesion presenting with a different frequency in psoriatic patients with or without arthralgia (16), thus it might represent an interesting feature to assess also in IBD.

A further limitation of this review can be represented by potential evolutions in the field of ultrasound, since the data-driven validation of the definition of enthesitis is still ongoing, and the lesions included in the definition are frequently detected also in healthy subjects (38). The concept of ultrasonographic enthesitis might therefore change in the future, implying a different interpretation of our results (39). The development of new biological drugs for IBD, moreover, may change the clinical panorama of these disorders (40).

The absence of follow-up studies precluded the evaluation of the long-term prognostic role of ultrasound abnormalities over the occurrence of joint manifestations in IBD patients who do not show any joint involvement. The main difficulty in this field is related to the low incidence of inflammatory arthritis in patients with IBD, and the study of such process would require large samples, observed for a very long follow-up. This reduces the feasibility of valid prognostic studies. Moreover, a recent SLR has underpinned several methodological issues in existing cohorts of IBD and SpA, requiring a further effort in achieving a standardized assessment (41). In addition to this, the data we obtained derive from studies conducted on treated IBD patients, in which some drugs might have masked the possible joint involvement.

The implications for clinical practice of our results include the necessity of prioritizing accurate clinical assessment in patients with IBD, particularly at early stages, in order to timely detect a potential joint involvement, determining a decreased quality of life and the potential development of disability. In this setting, musculoskeletal ultrasonography can represent a valid complementary and easily available imaging technique to support clinical evaluation in the outpatient setting. Our work highlighted several existing gaps in the literature on this topic, and in particular the urge for future prospective studies (Research Agenda, Table 4), in order to identify clinical and imaging predictors of arthritis in patients with IBD without overt joint involvement.

**Table 4:** Research Agenda of US in IBD without a diagnosis of arthritis.

1. To evaluate the diagnostic performance of a combination of different entheseal lesions to identity patients with IBD and arthritis
2. To further assess the frequency and diagnostic value of tenosynovitis and synovitis
3. To investigate the prognostic value of ultrasound in identifying IBD patients at risk of developing arthritis by large prospective studies
4. To produce evidence on the value of ultrasound in monitoring joint involvement
5. To explore the value of ultrasound in specific populations (early disease, treatment with biological drugs)

## Data Availability Statement

The original contributions presented in the study are included in the article/Supplementary Material, further inquiries can be directed to the corresponding author.

## Author Contributions

GS, AS, FB, and CM contributed to conception and design of the study. GS performed the searches and organized the database. AS, DS, FL, and EF performed the screening of the abstracts and the extraction of the results. GS and AS wrote the first draft of the manuscript. All authors contributed to manuscript revision, read, and approved the submitted version.

## Funding

This work was partially supported by the Ricerca Corrente funding scheme of the Ministry of Health, Italy.

## Conflict of Interest

The authors declare that the research was conducted in the absence of any commercial or financial relationships that could be construed as a potential conflict of interest.

## Publisher's Note

All claims expressed in this article are solely those of the authors and do not necessarily represent those of their affiliated organizations, or those of the publisher, the editors and the reviewers. Any product that may be evaluated in this article, or claim that may be made by its manufacturer, is not guaranteed or endorsed by the publisher.
