# Supplementary material for: Musculoskeletal Ultrasound to Identify Subclinical Joint and Periarticular Involvement in Patients With Inflammatory Bowel Disease: A Systematic Literature Review
Source: Front Med (Lausanne). 2022 May 16;9:919521. doi: 10.3389/fmed.2022.919521 (PMC9149094; doi:10.3389/fmed.2022.919521)
Supplement: Supplementary file 1 [file Table_1.docx]

**MUSCULOSKELETAL ULTRASOUND TO IDENTIFY SUBCLINICAL JOINT AND PERIARTICULAR INVOLVEMENT IN PATIENTS WITH INFLAMMATORY BOWEL DISEASE: A SYSTEMATIC LITERATURE REVIEW**

**Table S1:** Search strategies

| PubMed | #1 "Inflammatory Bowel Diseases"[Mesh]  #2 "Crohn Disease"[Mesh]  #3 "Colitis, Ulcerative"[Mesh]  #4 "Inflammatory bowel disease"  #5 "inflammatory bowel diseases"  #6 "Crohn" AND "disease"  #7 "ulcerative" AND "colitis"  #8 "regional enteritis"  #9 "ileitis"  #10 "colitis"  #11 "proctosigmoiditis"  #12 "rectocolitis"  #13 "rectosigmoiditis"  #14 "ulcerative proctocolitis"  #15 "haemorrhagic proctocolitis"  #16 OR 1-15  #17 "ultrasonography"[MeSH Terms]  #18 "ultrason*"[All Fields]  #19 "Ultrasound"[All Fields]  #20 "sonograph*"[All Fields]  #21 "ecograph*"[All Fields]  #22 "echotomograph*"[All Fields]  #23 "ultrasonograph*"[All Fields]  #24 # 16 AND #23  Limited to Humans, English, Adult, 1980-2021 |
| --- | --- |
| Embase | #1'echography'/exp OR 'echography'  #2ultrasonograph*  #3'ultrasound'  #4#1 OR #2 OR #3  #5'inflammatory bowel disease'/exp OR 'inflammatory bowel disease' OR 'crohn disease'/exp OR 'crohn disease' OR 'ulcerative colitis'/exp OR 'ulcerative colitis'  #6'crohn' AND 'disease'  #7'ulcerative' AND 'colitis'  #8'regional enteritis'  #9'ileitis'  #10'colitis'  #11'proctosigmoiditis'  #12'rectocolitis'  #13'rectosigmoiditis'  #14'ulcerative proctocolitis'  #15'haemorrhagic proctocolitis'  #16'proctitis'  #17 OR 5-16  #18 #4 AND #17  Limited to Humans, English, Adult, Embase, 1980-2021 |

***Table S2. Summary of findings:*** Frequency of abnormalities detected by ultrasonography in patients with IBD without a diagnosis of arthritis. IBD: inflammatory bowel disease; CD: Crohn’s Disease; UC: ulcerative colitis; HC: healthy control; MTX: methotrexate; AZA: azathioprine; SSZ: sulphasalazine; TNF: tumor necrosis factor; PDN: prednisone; PD: power Doppler; GS: grey scale; GUESS: Glasgow Ultrasound Enthesitis Scoring System; MASEI: Madrid sonography enthesitis index; R: right; L: left; SpA: spondyloarthritis; OMERACT: outcome measures in rheumatology; MCP: metacarpophalangeal; MTP: metatarsophalangeal.

| **Study** | **N** | **Population** | **Study design** | **Intervention (US technique)** | **Comparator** | **Results** |
| --- | --- | --- | --- | --- | --- | --- |
| Bandinelli 2010 (1) | 81 IBD (55 CD and 26 UC)  40 HC | Inactive or low active IBD patients without a diagnosis of arthritis.  Disease duration 8.8 years  Treatment: MTX (3/81), AZA (2/81) and anti-TNF-α (8/81) | Case-control | LOGIQ5 General Electric 10-MHz  GUESS score (0-36)  Quadriceps tendon, proximal rotuleus, distal rotuleus, Achilles tendon and plantar fascia  For entheses thickness: Balint cut-off for quadriceps >6.1, proximal and distal rotuleus >4mm, Achilles >5.29, plantar fascia >4.4 mm.  For vascularity: binary, graded: no flow (Grade 0); mild (Grade 1); moderate (Grade 2); severe (more than three spots) (Grade 3).  Total PD: calculated by summing semi-quantitative PD scores of each tendon. | n.a. | ***Total***  Thickness: IBD 81.5%; controls 0%  Enthesophytes: IBD 67.9%; controls 5%  Bursitis: IBD 27.1%, controls 0%  Erosions: IBD 16%; controls 0%  PD: IBD 16%; controls 0%  ***Quadriceps***  Increased thickness R 34.5%, L 25.9%  Enthesophytes R 38.2%, L 4.9%  Bursitis R 0%, L 0%  Erosions R 2.5%, L 0%  PD R 2.5%, L 4.9%  ***Proximal patellar***  Increased thickness R 42%, L 33.3%  Enthesophytes R 2.4%, L 2.4%  Bursitis R 0%, L 0%  Erosions R 3.7%, L 0%  PD R 0%, L 3.7%  ***Distal patellar***  Increased Thickness R 58%, L 59.2%  Enthesophytes R 7.4%, L 4.9%  Bursitis R 21%, L 17.2%  Erosions R 3.7%, L 3.7%  PD R 3.7%, L 1.2%  ***Achilles tendon***  Increased thickness R 9.9%, L 9.9%  Enthesophytes R 56.8%, L 42%  Bursitis R 7.4%, L 6.1%  Erosions R 1.2%, L 1.2%  PD R 0%, L 4.9%  ***Plantar fascia***  Increased Thickness R 16%, L 13.6%  Enthesophytes R 3.7%, L 6.1%  Bursitis R 0%, L 0%  Erosions R 2.5%, L 1.2%  PD R 1.2%, L 4.9% |
| Bertolini 2020 (2) | 148 IBD  (68 CD, 77 UC) | Consecutive patients with IBD (CD and UC)  Treatment: 27/148 on biologics | Cross-sectional | ESAOTE MyLab70 or MyLabClass C, 18 MHz and 13 MHz probe  Common extensor tendon, quadriceps tendon, patellar tendon, tibial tuberosity, Achilles tendon and plantar fascia  MASEI and GUESS  Knees and ankles: synovial hypertrophy, effusion and articular erosions recorded as present or absent; the flexor and extensor tendons of the feet were evaluated for the presence synovial hypertrophy and fluid distension and PD | n.a. | ***Patient level***  Entheseal abnormalities: 87.8%  PD + enthesitis: 27.1%  Acute entheseal abnormalities: 43.8%  Chronic entheseal abnormalities: 83.8%  Joint abnormalities: 19.7% |
| Hsiao 2014 (3) | 14 IBD  14 HC | Definite diagnosis of IBD (CD and UC)  Treatment: PDN 1, TNFi 1,Mesalazine/SSZ 14 | Case-control | GE LOGIQ e, 5-13 MHz  PD positive within 2 mm to the bony insertion  GUESS  Five entheseal sites: superior and inferior poles of the patella, tibial tuberosity, Achilles tendon, plantar aponeurosis. | n.a. | ***Entheseal level: all percentages refer to the number of entheses***  ***Total***  IBD: 42/140 (30%) abnormal sites  HC: 21/140 (15%) abnormal sites  ***Quadriceps***  *Thickening*  IBD: 8/140 (5.71&)  HC: 3/140 (2.14%)  *Bursitis*  IBD: 11/140 (7.86%)  HC: 9/140 (6.43%)  *Entesophytes*  IBD: 1/140 (0.71%)  HC: 0/140 (0%)  *Bone erosions*  IBD: 0/140 (0%)  HC: 0/140 (0%)  ***Superior patellar***  *Thickening*  IBD: 12/140 (8.57%)  HC: 9/140 (6.43%)  *Bursitis*  IBD: 0/140 (0%)  HC: 0/140 (0%)  *Entesophytes*  IBD: 0/140 (0%)  HC: 0/140(0%)  *Bone erosions*  IBD: 0/140 (0%)  HC: 0/140 (0%)  ***Inferior patellar***  *Thickening*  IBD: 9/140 (6.43%)  HC: 2/140 (1.43%)  *Bursitis*  IBD: 0/140 (0%)  HC: 0/140 (0%)  *Entesophytes*  IBD: 0/140 (0%)  HC: 0/140 (0%)  *Bone erosions*  IBD: 0/140 (0%)  HC: 0/140 (0%)  ***Achilles tendon***  *Thickening*  IBD: 1/140 (0.71%)  HC: 0/140 (0%)  *Bursitis*  IBD: 3/140 (2.14%)  HC: 0/140 (0%)  *Entesophytes*  IBD: 2/140 (1.42%)  HC: 0/140 (0%)  *Bone erosions*  IBD: 0/140 (0%)  HC: 0/140 (0%)  ***Plantar fascia***  *Thickening*  IBD: 0/140 (0%)  HC: 0/140 (0%)  *Bursitis*  IBD: 0/140 (0%)  HC: 0/140 (0%)  *Entesophytes*  IBD: 0/140 (0%)  HC: 0/140 (0%)  *Bone erosions*  IBD: 0/140 (0%)  HC: 0/140 (0%) |
| Husic 2021(4) | 47 IBD patients (CD and UC, 33 without SpA)  44 HC | Adults with IBD  Median disease duration in years: without SpA: 10; with SpA: 7.5 | Case-control | Esaote MyLab Twice, 18-MHz  GS and PD  14 entheses: bilateral triceps, lateral epicondyles, distal insertion of quadriceps, proximal and distal insertion of patellar tendon, distal insertion of Achilles tendon and plantar fascia  MASEI  Modified MASEI on 14 entheses | n.a. | ***IBD pts without SpA***  **Positive PD**  22 pts (67%)  Highest prevalence at lateral epicondyle (67% of all PD positive enthesis)  **Erosions**  18 pts (15%)  Higher prevalence at the distal insertion of patellar tendon (23% of all erosions), triceps tendon (20%) and Achilles tendon (19%) |
| Martinis 2020(5) | 158 IBD, of which 98 without SpA | Adults with IBD  Mean disease duration (months, sd) 116 (117) | Cross-sectional | Esaote My Lab Twice,18–6 and 13–5MHz  PD <2mm from the boy surface  Enthesitis was defined according to the OMERACT definition.  Bilaterally assessment of common extensor tendon insertion, superior and inferior pole of the patella, tibial tuberosity, plantar fascia and Achilles tendon.  MASEI and GUESS | n.a. | ***Patients with >1 entheseal abnormality***  75/98 (87%)  ***Patients with >1 acute entheseal change***  30/98 (31%)  ***Patients with>1 enthesis with chronic changes***  81/98 (83%)  ***Patients with>1 enthesis with PD***  14/98 (14%) |
| Rodriguez-Caminero 2020(6) | 112 IBD without SpA  Paired HC | Adults with IBD | Cross-sectional | GE Logiq 5 PRO; 7–12 MHz.  GS and PD  12 peripheral joints and 12 entheses: bilateral lateral epicondyle, medial epicondyle, quadriceps tendon, proximal patellar tendon, distal patellar tendon, Achilles tendon, and plantar fascia. | n.a. | **Active enthesitis and/or synovitis**  40.2% |
| Rovisco 2016(7) | 76 IBD (43 CD, 33 UC)  20 SpA  45 HC | IBD without musculoskeletal symptoms | Cross-sectional | MyLab Twice, MyLab 70XVG, GE LOGIQ P5; 6-18 MHz.  Bilateral MCP 2-3, knees, ankles, MTP 1 joints, quadriceps, distal and proximal patellar, Achilles, and plantar aponeurosis entheseal insertion.  GS and PD  OMERACT definition of entheseal abnormalities | n.a. | ***Patient level – any abnormality***  ***All joints***  21/76 (48.8%)  ***MCPs***  3/76 (5.2%)  ***Knee***  12/76 (15.8%)  ***Ankle***  7/76 (11.3%)  ***MTP I***  17/76 (22.4%) |
| Ureyen 2018(8) | 43 IBD (12 CD, 31 UC) | IBD with and without musculoskeletal symptoms  Disease duration 70.79 (81.5) months | Cross-sectional | GE LOGIQ P9, 7-13MHz  Bilateral quadriceps tendon, proximal and distal patellar tendon, Achilles tendon, plantar aponeurosis and triceps tendon were examined bilaterally,  Hypoechogenicity (0-3), thickening (quantitative), enthesophytes (0-3), calcifications (0-3), erosions (quantitative) and PD (0-3)  Inflammation and damage score  GS and PD | n.a. | ***Site level***  ***Quadriceps***  Hypoechogenicity 9/86 (10.5%)  Thickening 35/86 (40.7%)  PD 2/86 (2.3%)  Entesophyte 0/86 (0%)  Calcification 0/86 (0%)  Erosion 2/86 (2.3%)  ***Proximal patellar***  Hypoechogenicity 4/86 (4.7%)  Thickening 56/86 (65.1%)  PD 3/86 (3.5%)  Entesophyte 1/86 (1.2%)  Calcification 1/86 (1.2%)  Erosion 2/86 (2.3%)  ***Distal patellar***  Hypoechogenicity 17/86 (19.8%)  Thickening 42/86 (48.8%)  PD 14/86 (16.3%)  Entesophyte 0/86 (0%)  Calcification 1/86 (1.2%)  Erosion 0/86 (0%)  ***Achilles tendon***  Hypoechogenicity 29/86 (33.7%)  Thickening 20/86 (23.3%)  PD 18/86 (20.9%)  Entesophyte 17/86 (19.8%)  Calcification 2/86 (2.3%)  Erosion 1/86 (1.2%)  ***Plantar aponeurosis***  Hypoechogenicity 1 (1.2%)  Thickening 7 (8.1%)  PD 1 (1.2%)  Entesophyte 0/86 (0%)  Calcification 0/86 (0%)  Erosion 0/86 (0%)  ***Triceps tendon***  Hypoechogenicity 0/86 (0%)  Thickening 63/86 (73.3%)  PD 0/86 (0%)  Entesophyte 0/86 (0%)  Calcification 0/86 (0%)  Erosion 0/86 (0%) |

***Table S3: Summary of findings:*** Frequency of abnormalities detected by ultrasonography in patients with IBD without a diagnosis of arthritis, comparison of Crohn’s disease and ulcerative colitis. IBD: inflammatory bowel disease; CD: Crohn’s Disease; UC: ulcerative colitis; HC: healthy control; MTX: methotrexate; AZA: azathioprine; TNF: tumor necrosis factor; PD: power Doppler; GS: grey scale; GUESS: Glasgow Ultrasound Enthesitis Scoring System; MASEI: Madrid sonography enthesitis index; SpA: spondyloarthritis; OMERACT: outcome measures in rheumatology.

| **Study** | **N** | **Population** | **Study design** | **Intervention (US technique)** | **Comparator** | **Results** |
| --- | --- | --- | --- | --- | --- | --- |
| Bertolini 2020 (2) | 148 IBD  (68 CD, 77 UC) | Consecutive patients with IBD (CD and UC)  Treatment: 27/148 on biologics | Cross-sectional | ESAOTE MyLab70 or MyLabClass C, 18 MHz and 13 MHz probe  Common extensor tendon, quadriceps tendon, patellar tendon, tibial tuberosity, Achilles tendon and plantar fascia  MASEI and GUESS  Knees and ankles: synovial hypertrophy, effusion and articular erosions recorded as present or absent; the flexor and extensor tendons of the feet were evaluated for the presence synovial hypertrophy and fluid distension and PD | n.a. | ***At least one altered enthesis***  CD: 57/68 (83.8%) vs UC: 70/77 (90.2%) (ns)  ***At least one PD positive enthesitis***  CD: 14/68 (21.5%) vs UC: 24/77 (31.6%) (ns)  ***At least one acute entheseal abnormality***  CD: 28/68 (42.4%) vs UC: 34/77 (45.3%) (ns)  ***At least one chronic entheseal abnormality***  CD: 54/68 (79.4%) vs UC: 67/77 (87%) (ns)  ***At least one joint with abnormalities***  CD: CD: 10 (14.7%) vs 18 (23.7%) (ns) |
| Bandinelli 2010 (1) | 81 IBD (55 CD and 26 UC)  40 HC | Inactive or low active IBD patients without a diagnosis of arthritis.  Disease duration 8.8 years  Treatment: MTX (3/81), AZA (2/81) and anti-TNF-α (8/81) | Case-control | LOGIQ5 General Electric 10-MHz  GUESS score (0-36)  Quadriceps tendon, proximal rotuleus, distal rotuleus, Achilles tendon and plantar fascia  For entheses thickness: Balint cut-off for quadriceps >6.1, proximal and distal rotuleus >4mm, Achilles >5.29, plantar fascia >4.4 mm.  For vascularity: binary, graded: no flow (Grade 0); mild (Grade 1); moderate (Grade 2); severe (more than three spots) (Grade 3).  Total PD: calculated by summing semi-quantitative PD scores of each tendon. | n.a. | There was no difference in GUESS and PD between CD and UC. |
| Rovisco 2016 | 76 IBD (43 CD, 33 UC)  20 SpA  45 HC | IBD without musculoskeletal symptoms | Cross-sectional | MyLab Twice, MyLab 70XVG, GE LOGIQ P5; 6-18 MHz.  Bilateral MCP 2-3, knees, ankles, MTP 1 joints, quadriceps, distal and proximal patellar, Achilles, and plantar aponeurosis entheseal insertion.  GS and PD  OMERACT definition of entheseal abnormalities | n.a. | ***Patient level – any abnormality***  ***All joints***  CD 11/43 (25.5%) vs UC 10/33 (30.3%) (ns)  ***MCPs***  CD 3/43 (6.9%) vs UC 0/33 (0%) (ns)  ***Knee***  CD 4/43 (9.3%) vs UC 8/33 (24.2%) (ns)  ***Ankle***  CD 4/43 (9.3%) vs UC 3/33 (9.1%) (ns)  ***MTP I***  CD 11/43 (25.5%) vs UC 3/33 (9.1%) (ns) |

***Table S4: Summary of findings:*** Frequency of abnormalities detected by ultrasonography in patients with IBD without a diagnosis of arthritis, comparison of active and inactive IBD. IBD: inflammatory bowel disease; CD: Crohn’s Disease; UC: ulcerative colitis; HC: healthy control; MTX: methotrexate; AZA: azathioprine; TNF: tumor necrosis factor; PD: power Doppler; GS: grey scale; GUESS: Glasgow Ultrasound Enthesitis Scoring System; MASEI: Madrid sonography enthesitis index; CDAI: Crohn Disease Activity Index.

| **Study** | **N** | **Population** | **Study design** | **Intervention (US technique)** | **Comparator** | **Results** |
| --- | --- | --- | --- | --- | --- | --- |
| Husic 2021(4) | 47 IBD patients (CD and UC, 33 without SpA)  44 HC | Adults with IBD  Median disease duration in years: without SpA: 10; with SpA: 7.5 | Case-control | Esaote MyLab Twice, 18-MHz  GS and PD  14 entheses: bilateral triceps, lateral epicondyles, distal insertion of quadriceps, proximal and distal insertion of patellar tendon, distal insertion of Achilles tendon and plantar fascia  MASEI  Modified MASEI on 14 entheses | n.a. | No association was found between clinical IBD activity (CDAI and partial Mayo score) and MASEI, nor between clinical IBD activity and erosion-, PD- and enthesophyte subscores. |
| Bandinelli 2010 (1) | 81 IBD (55 CD and 26 UC)  40 HC | Inactive or low active IBD patients without a diagnosis of arthritis.  Disease duration 8.8 years  Treatment: MTX (3/81), AZA (2/81) and anti-TNF-a (8/81) | Case-control | LOGIQ5 General Electric 10-MHz  GUESS score (0-36)  Quadriceps tendon, proximal rotuleus, distal rotuleus, Achilles tendon and plantar fascia  For entheses thickness: Balint cut-off for quadriceps >6.1, proximal and distal rotuleus >4mm, Achilles >5.29, plantar fascia >4.4 mm.  For vascularity: binary, graded: no flow (Grade 0); mild (Grade 1); moderate (Grade 2); severe (more than three spots) (Grade 3).  Total PD: calculated by summing semi-quantitative PD scores of each tendon. | n.a. | There was no difference in GUESS and PD between IBD remittent (46 CD and 22 UC) and low active (9 CD and 4 UC). |

***Table S5: Summary of findings:*** Frequency of abnormalities detected by ultrasonography in patients with IBD without a diagnosis of arthritis, comparison based on disease duration. IBD: inflammatory bowel disease; CD: Crohn’s Disease; UC: ulcerative colitis; HC: healthy control; MTX: methotrexate; AZA: azathioprine; TNF: tumor necrosis factor; PD: power Doppler; GS: grey scale; GUESS: Glasgow Ultrasound Enthesitis Scoring System; MASEI: Madrid sonography enthesitis index; NS: not significant.

| **Study** | **N** | **Population** | **Study design** | **Intervention (US technique)** | **Comparator** | **Results** |
| --- | --- | --- | --- | --- | --- | --- |
| Bandinelli 2010 (1) | 81 IBD (55 CD and 26 UC)  40 HC | Inactive or low active IBD patients without a diagnosis of arthritis.  Disease duration 8.8 years  Treatment: MTX (3/81), AZA (2/81) and anti-TNF-a (8/81) | Case-control | LOGIQ5 General Electric 10-MHz  GUESS score (0-36)  Quadriceps tendon, proximal rotuleus, distal rotuleus, Achilles tendon and plantar fascia  For entheses thickness: Balint cut-off for quadriceps >6.1, proximal and distal rotuleus >4mm, Achilles >5.29, plantar fascia >4.4 mm.  For vascularity: binary, graded: no flow (Grade 0); mild (Grade 1); moderate (Grade 2); severe (more than three spots) (Grade 3).  Total PD: calculated by summing semi-quantitative PD scores of each tendon. | n.a. | GUESS and tPD did not correlate with disease duration. |
| Bertolini 2020 (2) | 148 IBD  (68 CD, 77 UC) | Consecutive patients with IBD (CD and UC)  Treatment: 27/148 on biologics | Cross-sectional | ESAOTE MyLab70 or MyLabClass C, 18 MHz and 13 MHz probe  Common extensor tendon, quadriceps tendon, patellar tendon, tibial tuberosity, Achilles tendon and plantar fascia  MASEI and GUESS  Knees and ankles: synovial hypertrophy, effusion and articular erosions recorded as present or absent; the flexor and extensor tendons of the feet were evaluated for the presence synovial hypertrophy and fluid distension and PD | n.a. | ***At least one altered enthesis***  >12 months 85/98 (90%) vs <12 months 38/53 (72%), p = 0.003  ***At least one enthesis with erosions***  >12 months 7/98 (7.4%) vs <12 months 0/53 (0%), p = 0.04  ***GUESS (mean,sd)***  >12 months 5.2 (3.5) versus 5.0 (3.8) , p = NS  ***MASEI (mean,sd)***  >12 months 5.2 (3.5) versus 5.0 (3.8) , p =NS |
| Ureyen 2018 | 43 IBD (12 CD, 31 UC) | IBD with and without musculoskeletal symptoms  Disease duration 70.79 (81.5) months | Cross-sectional | GE LOGIQ P9, 7-13MHz  Bilateral quadriceps tendon, proximal and distal patellar tendon, Achilles tendon, plantar aponeurosis and triceps tendon were examined bilaterally,  Hypoechogenicity (0-3), thickening (quantitative), enthesophytes (0-3), calcifications (0-3), erosions (quantitative) and PD (0-3)  Inflammation and damage score  GS and PD | n.a. | Disease duration was independent of inflammation and damage scores. |

***Table S6: Summary of findings:*** value of ultrasonographic findings in making a diagnosis of arthritis in patients with IBD without a diagnosis of arthritis. IBD: inflammatory bowel disease; CD: Corhn’s disease; UC: ulcerative colitis; MASEI: Madrid sonography enthesitis index; GUESS: Glasgow Ultrasound Enthesitis Scoring System; Se: sensitivity; Sp: specificity; LR+: positive likelihood ratio; LR-: negative likelihood ratio; PPV: positive predictive value; NPV: negative predictive value; GS: grey scale; PD: power Doppler; SpA: spondyloarthritis.

| **Study** | **N** | **Population** | **Study design** | **Intervention (US technique)** | **Reference standard** | **Results** |
| --- | --- | --- | --- | --- | --- | --- |
| Bertolini 2020(2) | 148 IBD  (68 CD, 77 UC) | Consecutive patients with IBD (CD and UC)  27/148 on biologics | Cross-sectional | ESAOTE MyLab70 or MyLabClass C, 18 MHz and 13 MHz probe  Common extensor tendon, quadriceps tendon, patellar tendon, tibial tuberosity, Achilles tendon and plantar fascia  MASEI and GUESS  Knees and ankles: synovial hypertrophy, effusion and articular erosions recorded as present or absent; the flexor and extensor tendons of the feet were evaluated for the presence synovial hypertrophy and fluid distension and PD | ASAS criteria | **At least one joint with US abnormalities**  Se (95%CI) 0.32 (0.20,0.46)  Sp (95%CI) 0.88 (0.79,0.94)  LR+ (95%CI) 2.69 (1.37,5.27)  LR- (95% CI) 0.77 (0.63,0.94)  PPV (95%CI) 0.62 (0.45,0.76)  NPV (95%CI) 0.68 (0.64,0.72)  **At least one enthesis with US abnormalities**  Se (95%CI) 0.87 (0.76,0.95)  Sp (95%CI) 0.15 (0.08,0.24)  LR+ (95%CI) 1.03 (0.9,1.18)  LR- (95% CI) 0.82 (0.35,1.91)  PPV (95%CI) 0.38 (0.35,0.42)  NPV (95%CI) 0.66 (0.46,0.82)  **At least one chronic entheseal abnormality**  Se (95%CI) 0.86 (0.74,0.97)  Sp (95%CI)0.17 (0.10,0.27)  LR+ (95%CI) 1.04 (0.9,1.20)  LR- (95% CI) 0.82 (0.38,1.79)  PPV (95%CI) 0.39 (0.35,0.42)  NPV (95%CI) 0.66 (0.47,0.81)  **At least one acute entheseal abnormality**  Se (95%CI) 0.59 (0.45,0.72)  Sp (95%CI) 0.65 (0.55,0.75)  LR+ (95%CI) 1.69 (1.19,2.42)  LR- (95% CI) 0.63 (0.44,0.89)  PPV (95%CI) 0.51 (0.42,0.59)  NPV (95%CI) 0.72 (0.65,0.79)  **At least one enthesis with PD**  Se (95%CI) 0.52 (0.38,0.65)  Sp (95%CI) 0.87 (0.78,0.93)  LR+ (95%CI) 3.97 (2.21,7.13)  LR- (95% CI) 0.55 (0.42,0.74)  PPV (95%CI) 0.70 (0.57,0.81)  NPV (95%CI) 0.74 (0.69,0.79) |
| Husic 2021(4) | 47 IBD patients (CD and UC, 33 without SpA)  44 HC | Adults with IBD  Median disease duration in years: without SpA: 10; with SpA: 7.5 | Case-control | Esaote MyLab Twice, 18-MHz  GS and PD  14 entheses: bilateral triceps, lateral epicondyles, distal insertion of quadriceps, proximal and distal insertion of patellar tendon, distal insertion of Achilles tendon and plantar fascia  MASEI  Modified MASEI on 14 entheses | Clinical diagnosis | **Entheseal PD**  Se (95%CI) 0.64 (0.35,0.87)  Sp (95%CI) 0.33 (0.18,0.52)  LR+ (95%CI) 0.96 (0.61,1.53)  LR- (95% CI) 1.07 (0.46,2.51)  PPV (95%CI) 0.29 (0.17,0.45)  NPV (95%CI) 0.69 (0.48,0.84)  **Entheseal erosions**  Se (95%CI) 0.79 (0.49,0.95)  Sp (95%CI) 0.45 (0.28,0.64)  LR+ (95%CI) 1.44 (0.95,2.18)  LR- (95% CI) 0.47 (0.16,1.37)  PPV (95%CI) 0.38 (0.29,0.48)  NPV (95%CI) 0.83 (0.63,0.94) |
| Martinis 2020(5) | 158 IBD, of which 98 without SpA | Adults with IBD  disease duration (months, sd) 116 (117) | Cross-sectional | Esaote My Lab Twice,18–6 and 13–5MHz  PD <2mm from the bony surface  Enthesitis was defined according to the OMERACT definition.  Bilaterally assessment of common extensor tendon insertion, superior and inferior pole of the patella, tibial tuberosity, plantar fascia and Achilles tendon.  MASEI and GUESS | ASAS criteria | **At least one acute change**  Se (95% CI) 0.61 (0.45,0.75)  Sp (95% CI) 0.67 (0.57,0.76)  LR+ (95% CI) 1.84 (1.30,2.62)  LR- (95% CI) 0.58 (0.40,0.86)  PPV (95% CI) 0.43 (0.35,0.52)  NPV (95% CI) 0.80 (0.74,0.86)  **At least one chronic change**  Se (95% CI) 0.76 (0.62,0.83)  Sp (95% CI) 0.17 (0.10,0.25)  LR+ (95% CI) 0.92 (0.77,1.09)  LR- (95% CI) 1.41 (0.75,2.69)  PPV (95% CI) 0.29 (0.25,0.32)  NPV (95% CI) 0.61 (0.45,0.75)  **PD**  Se (95% CI) 0.56 (0.41,0.71)  Sp (95% CI) 0.84 (0.76,0.90)  LR+ (95% CI) 3.75 (2.25,6.18)  LR- (95% CI) 0.51 (0.37,0.73)  PPV (95% CI) 0.60 (0.48,0.71)  NPV (95% CI) 0.82 (0.77,0.87) |

**References**

1. Bandinelli F, Milla M, Genise S, Giovannini L, Bagnoli S, Candelieri A, et al. Ultrasound discloses entheseal involvement in inactive and low active inflammatory bowel disease without clinical signs and symptoms of spondyloarthropathy. Rheumatol Oxf Engl. luglio 2011;50(7):1275–9.

2. Bertolini E, Macchioni P, Rizzello F, Salice M, Vukatana G, Sandri G, et al. Ultrasonographic and clinical assessment of peripheral enthesitis and arthritis in an Italian cohort of inflammatory bowel disease patients. Semin Arthritis Rheum. giugno 2020;50(3):436–43.

3. Hsiao Y-F, Wei S-C, Lu C-H, Wu C-H, Hsieh S-C, Li K-J. Patients with inflammatory bowel disease have higher sonographic enthesitis scores than normal individuals: Pilot study in Taiwan. J Med Ultrasound. 2014;22(4):194–9.

4. Husic R, Lackner A, Kump PK, Högenauer C, Graninger W, Dejaco C. High Prevalence of Ultrasound Verified Enthesitis in Patients With Inflammatory Bowel Disease With or Without Spondylarthritis. Front Med [Internet]. 2021;8((Husic R.; Lackner A.; Graninger W.; Dejaco C., christian.dejaco@gmx.net) Department of Rheumatology and Immunology, Medical University Graz, Graz, Austria). Disponibile su: https://www.embase.com/search/results?subaction=viewrecord&id=L634307601&from=export

5. Martinis F, Tinazzi I, Bertolini E, Citriniti G, Variola A, Geccherle A, et al. Clinical and sonographic discrimination between fibromyalgia and spondyloarthopathy in inflammatory bowel disease with musculoskeletal pain. Rheumatol Oxf Engl. 1 ottobre 2020;59(10):2857–63.

6. Rodríguez-Caminero S, Queiro R. Ultrasound subclinical musculoskeletal findings in inflammatory bowel disease: diagnostic value of positive Doppler signal. Rheumatol Oxf Engl. 1 novembre 2020;59(11):3571–2.

7. Rovisco J, Duarte C, Batticcioto A, Sarzi-Puttini P, Dragresshi A, Portela F, et al. Hidden musculoskeletal involvement in inflammatory bowel disease: a multicenter ultrasound study. BMC Musculoskelet Disord. 16 febbraio 2016;17:84.

8. Bakirci Ureyen S, Karacaer C, Toka B, Erturk Z, Eminler AT, Kaya M, et al. Similar subclinical enthesitis in celiac and inflammatory bowel diseases by ultrasound suggests a gut enthesis axis independent of spondyloarthropathy spectrum. Rheumatol Oxf Engl. 1 agosto 2018;57(8):1417–22.
